# Current Status of Radiopharmaceuticals for the Theranostics of Neuroendocrine Neoplasms

**DOI:** 10.3390/ph10010030

**Published:** 2017-03-15

**Authors:** Melpomeni Fani, Petra Kolenc Peitl, Irina Velikyan

**Affiliations:** 1 Division of Radiopharmaceutical Chemistry, University Hospital of Basel, 4031 Basel, Switzerland; melpomeni.fani@usb.ch; 2 Department of Nuclear Medicine, University Medical Centre Ljubljana, 1000 Ljubljana, Slovenia; petra.peitl@kclj.si; 3 Department of Medicinal Chemistry, Uppsala University, 751 23 Uppsala, Sweden; irina.velikyan@akademiska.se

**Keywords:** neuroendocrine neoplasms, theranostics, radiolabeled peptides, somatostatin receptor antagonists, GLP-1R, exendin-4, CCK2, gastrin, GIP

## Abstract

Nuclear medicine plays a pivotal role in the management of patients affected by neuroendocrine neoplasms (NENs). Radiolabeled somatostatin receptor analogs are by far the most advanced radiopharmaceuticals for diagnosis and therapy (radiotheranostics) of NENs. Their clinical success emerged receptor-targeted radiolabeled peptides as an important class of radiopharmaceuticals and it paved the way for the investigation of other radioligand-receptor systems. Besides the somatostatin receptors (sstr), other receptors have also been linked to NENs and quite a number of potential radiolabeled peptides have been derived from them. The Glucagon-Like Peptide-1 Receptor (GLP-1R) is highly expressed in benign insulinomas, the Cholecystokinin 2 (CCK2)/Gastrin receptor is expressed in different NENs, in particular medullary thyroid cancer, and the Glucose-dependent Insulinotropic Polypeptide (GIP) receptor was found to be expressed in gastrointestinal and bronchial NENs, where interestingly, it is present in most of the sstr-negative and GLP-1R-negative NENs. Also in the field of sstr targeting new discoveries brought into light an alternative approach with the use of radiolabeled somatostatin receptor antagonists, instead of the clinically used agonists. The purpose of this review is to present the current status and the most innovative strategies for the diagnosis and treatment (theranostics) of neuroendocrine neoplasms using a cadre of radiolabeled regulatory peptides targeting their receptors.

## 1. Introduction

Neuroendocrine neoplasms (NENs) originate from endocrine cells located in endocrine organs (pituitary, thyroid, pancreas, adrenal glands) or in disseminated endocrine tissues of the lung or along the intestinal tract. Neuroendocrine cells are characterized by neuroamine uptake mechanisms and by the presence of peptide receptors on their membrane. These features constitute the basis of the clinically used radiopharmaceuticals in NEN imaging and therapy [1,2,3]. A series of radiopharmaceuticals are used for metabolic imaging of NEN, such as the amine precursors ^18^F-DOPA, ^11^C-5-HTP, the noradrenalin analog ^123^I-MIBG and the glucose analog ^18^F-FDG for glucose metabolism. On the other hand, somatostatin receptor (sstr) imaging by means of scintigraphy, SPECT or PET/CT is by far the most advanced imaging approach for NENs, given the high expression of sstr on neuroendocrine cells. This is also the molecular basis for treatment of NENs using somatostatin analogs in the context of radiotheranostics [4,5].

**Figure 1:**
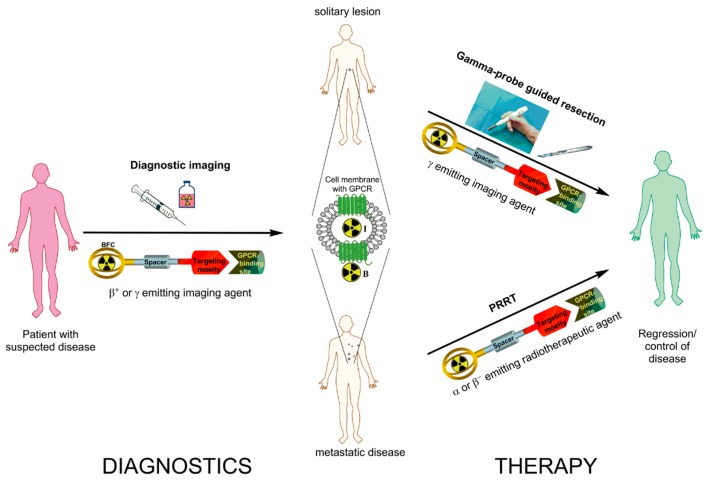
Schematic representation of radiotheranostics using radiolabeled peptides targeting G-protein coupled receptors (GPCR). The radiopharmaceutical consists of the targeting moiety (peptide), a bifunctional chelator (BFC) forming a stable complex with the radionuclide, and often a spacer in between the two units. Radiotheranostics involves diagnostic (left panel) and therapeutic (right panel) components. In targeting of GPCRs (somatostatin receptor (sstr), glucagon-like peptide-1 receptor (GLP-1R), cholecystokinin 2 receptor (CCK2R) or glucose-dependent insulinotropic polypeptide receptor (GIP-R); middle panel) the radiotheranostic agent would bind to GPCR and either internalize into the cell, when utilizing an agonist (I) or stay on the surface of the cell, when utilizing an antagonist (B). In the first step diagnostic imaging using β^+^ or γ emitting imaging agent to assess the spread of disease is conducted. On the therapeutic stage, in the given example, the patient is subjected either to the gamma-probe guided resection or Peptide Receptor Radionuclide Therapy (PRRT). In radioguided surgery pre-administration of corresponding γ emitting imaging agent gives real-time information to the surgeon and guides the localization of the GPCR rich tumor site intraoperatively. In the case of a metastatic disease α or β^−^ emitting radiotherapeutic agent is administered to target the GPCR rich lesions internally (PRRT).

Theranostics in its broader meaning embraces diagnostic methods conducted for the prediction of the efficacy of specific therapeutic interventions on individual basis as well as for the monitoring response to the treatment (Figure 1). When applied to nuclear medicine with the use of radioactive agents targeted at specific biological processes, it can be referred as radiotheranostics [6]. The targeted imaging in oncology provides tumor-type specific non-invasive diagnosis, precise localization of tumors and metastases that most importantly have the potential for pre-therapeutic quantification of receptor status, uptake kinetics and dosimetry that enables accurate therapy selection and planning, as well as monitoring response to the therapy resulting in personalized medicine.

As mentioned above the radiotheranostics, wherein the pre-therapeutic imaging and internal radiotherapy is conducted with the same vector molecule exchanging the imaging and therapeutic radionuclides, has been clinically realized targeting sstr in patients affected by NENs. Somatostatin analogs labeled with β^+^ emitting ^68^Ga for PET imaging and β^−^ emitting ^177^Lu or ^90^Y for the internal radiotherapy are most commonly used [5,7]. Ideally the pre-therapeutic imaging and subsequent radiotherapy should be conducted using the radioactive isotopes of the same chemical element, e.g., ^123,124^I/^131^I. However, it is not always possible to achieve in practice. Ga(III), Lu(III) and Y(III) form stable complexes with DOTA chelator, thus introducing the least possible modification to a ligand.

The success of sstr-targeting with radiolabeled somatostatin analogs lies on the fact that neuroendocrine cells express sstr in high incidence and density. Five somatostatin receptor subtypes are known (sstr1-sstr5) with sstr2 being the most prominent one in differentiated NENs [8], being also the primary target of current (non-radioactive) somatostatin analog therapy for NENs. The success also lies on the fact that regulatory peptides targeting G-protein coupled receptors (GPCRs), member of which is the sstr, is a class of high affinity ligands. These peptidic ligands can be chemically tuned to target the cell-surface receptors that are actually expressed in very high density on the cancer cells, compared to their relatively low density in physiological organs. This offers the possibility of accumulation of the radiolabeled peptides on the diseased tissue with minimal accumulation to neighboring, normal tissues. 

^111^In-Diethylenetriaminepentaacetic acid (DTPA)-octreotide (Octreoscan^TM^, Mallinckrodt, Maryland Heights, USA) has covered for many years the medical need of imaging NENs, however, several PET imaging probes with improved characteristics followed Octreoscan^TM^. [^68^Ga-DOTA,Tyr^3^]octreotide (^68^Ga-DOTA-TOC), [^68^Ga-DOTA,Tyr^3^,Thr^8^]octreotate (^68^Ga-DOTA-TATE) or [^68^Ga-DOTA,1-Nal^3^]octreotide (^68^Ga-DOTA-NOC) are used clinically, while the same conjugates are being used in Peptide Receptor Radionuclide Therapy (PRRT) when labeled with ^90^Y or ^177^Lu (^90^Y/^177^Lu-DOTA-TOC and ^177^Lu-DOTA-TATE) [9,10,11]. Among these radiopharmaceuticals, ^68^Ga-DOTA-TATE (NETSPOT™, Advanced Accelerator Applications, Saluggia, Italy) has received approval by the US Food and Drug Administration (FDA), ^68^Ga-DOTA-TOC has received Orphan Drug Designation, while the therapeutic agent ^177^Lu-DOTA-TATE (Lutathera^®,^ Advanced Accelerator Applications) is under FDA review.

Nowadays, additional receptor-ligand systems have been linked to NENs and quite a number of potential radiolabeled peptide analogs have been derived from them. These systems include, the Glucagon-Like Peptide-1 Receptor (GLP-1R) targeted with radiolabeled exendin analogs, the Cholecystokinin 2 (CCK2)/Gastrin receptor targeted with radiolabeled gastrin analogs and the Glucose-dependent Insulinotropic Polypeptide (GIP) receptor targeted with radiolabeled GIP analogs.

Thus the radiotheranostics using somatostatin analogs have entered clinical practice, while the investigation of similar potential for ligands targeting GLP-1R is in progress. Radiopharmaceuticals targeting CCK2 with clinical applicability is on its exploratory stage, while the development of radiopharmaceuticals targeting GIP-R is in its infancy. The review presents the current status of the development of radiopharmaceuticals targeting sstr, GLP-1R, CCK2R, and GIP-R, in particular addressing the following aspects:

I Despite the fact that sstr2-targeting is well established and clinically accepted, an alternative approach questions the dogma of using radiolabeled somatostatin receptor agonists and demonstrates advantages of antagonists. This is the focus of the respective section covering sstr-targeting.
II The investigation of the clinical relevance of imaging radiopharmaceuticals targeting GLP-1R has already been conducted in humans, and the exploration of the feasibility of respective radiotherapeutic agents is in progress preclinically. The focus of the respective section is the advances and shortcomings in clinical studies as well as research conducted to overcome those shortcomings in the context of radiotheranostics.
III The field of CCK2 receptors is not new, but clinical success is still pending mainly due to a number of limitations of the developed radiolabeled gastrin analogs. These limitations and different approaches to circumvent them are discussed in the CCK2 targeting section, together with the current clinical status in this field.
IV Development of radioligands targeting of GIP-R is a newly emerging and very promising field. The first advances in GIP receptor and respective ligand and radioligand exploration are presented in the corresponding section.

## 2. Somatostatin Receptor Antagonists

The radiolabeled somatostatin receptor antagonists represent the recent most favorable innovation in molecular imaging and targeted radionuclide therapy of NENs. All somatostatin analogs that are currently used in the clinic (i.e., DOTA-TOC, DOTA-TATE, DOTA-NOC, Table 1) are agonists inducing internalization of the receptor-ligand complex upon their binding to the receptor. For years, internalization was thought to be of high importance for high and long-lasting tumor uptake of a radiotracer. However, a number of recent observations have challenged this strategy. Antagonists may have characteristics other than those related to internalization that make their radiolabeled derivatives suitable tools for in vivo receptor targeting. Most relevant is the in vitro evidence that, under certain circumstances, antagonist radioligands may recognize a higher number of receptor-binding sites than agonist radioligands do [12,13,14]. The field of radiolabeled somatostatin receptor antagonists is novel and most of the data supporting their use are based on preclinical studies. In the meantime, pilot studies in humans and preliminary results of the first phase I/II clinical study with antagonists are also available and discussed herein.

### 2.1. Development of Radiopharmaceuticals Based on Somatostatin Receptor Antagonists

The groups of Maecke (University Hospital of Basel), Rivier (the Salk Institute) and Reubi (University of Bern), were the first to develop and study radiolabeled somatostatin receptor antagonists as potential radiopharmaceuticals [14]. They used the very first somatostatin-based antagonist, developed by Bass et al, with selectivity for the sstr2 (sst2-ANT or BASS (Table 1)) [15] and the antagonist sst3-ODN-8, with selectivity for the sstr3, developed by the group of Rivier [16]. Both antagonists were labeled with ^111^In via the chelator DOTA and evaluated in vivo using the HEK293 cell line stably transfected with the rat sstr2. The direct comparison of ^111^In-DOTA-BASS (IC_50_ = 9.4 ± 0.4 nM [14]) with the agonist ^111^In-DTPA-TATE (IC_50_ = 1.3±0.2 nM [17]) in HEK-sstr2 xenografts indicated very early that the antagonists may be superior to agonists for in vivo sstr2 targeting. The tumor uptake for ^111^In-DOTA-BASS was twice as high as for ^111^In-DTPA-TATE, despite its lower affinity and lack of internalization. The same was true for the ^111^In-DOTA-sst3-ODN-8 compared with ^111^In-DOTA-NOC when evaluated in rat HEK-sstr3 xenografts. The conclusion was also credible for the human receptors as no difference was seen when comparing the uptake and pharmacokinetics of ^111^In-DOTA-BASS in rat and human HEK-sstr2 xenografts [18]. Explanations for these excellent in vivo targeting properties of the antagonist may be found, at least in part, in the higher number of binding sites (B_max_) recognised by the antagonist, compared to agonist [14]. However, in another study by the Anderson group comparing BASS with TATE, both labeled with ^64^Cu via CB-TE2A [19], the antagonist did not show higher tumor uptake in vivo using the rat pancreatic cell line AR42J, despite the fact that a higher number of binding sites was also found for the antagonist.

The design of more potent sstr2-antagonists with improved affinity, compared to ^111^In-DOTA-BASS, followed [20], based on known structural features for converting an agonist into a potent antagonist (inversion of chirality at position 1 and 2 of the octreotide family) and on structure-activity-relationship studies [21]. Out of this series different radiolabeled sstr2-antagonists were developed and studied aiming firstly, to develop new radiotracers for imaging and therapy of NENs and secondly, to identify structural parameters determining the pharmacological properties of radiolabeled somatostatin receptor antagonists [22,23]. Based on the new sstr2-antagonist LM3 (Table 1) optium chelator-radiometal combinations were used for the development of four PET imaging agents: ^68^Ga-DOTA-LM3, ^68^Ga-NODAGA-LM3, ^64^Cu-NODAGA-LM3 and ^64^Cu-CB-TE2A-LM3 [22]. Their comparative preclinical evaluation demonstrated that the chelate defines affinity and also pharmacokinetics of the radiolabeled sstr2-antagonists. For instance, ^68^Ga-NODAGA-LM3 has a 10-fold higher sstr2-affinity than ^68^Ga-DOTA-LM3 and a 5-fold higher than the corresponding ^64^Cu-complex (^64^Cu-NODAGA-LM3). Comparing ^64^Cu-NODAGA-LM3 with ^64^Cu-CB-TE2A-LM3 the latter had almost no washout from the tumor up to 24 h (surprising for an non-internalized radioligand), in contrast to ^68^Ga-NODAGA-LM3 that was washout out by 60% within 24 h. However, the tumor-to-normal tissue ratios were remarkably higher for ^64^Cu-NODAGA-LM3 over time.

A comprehensive study including three different sstr2-antagonists, namely LM3, JR10 and JR11 (Table 1), conjugated to the chelators DOTA and NODAGA and complexed with various (radio)metals such as In(III), Y(III), Lu(III), Cu(II) and Ga(III) showed that all Ga-DOTA analogs were the least affine radiotracers. The sstr2 binding affinity of the Ga-DOTA analogs was up to 60 times lower than the respective Y(III)-DOTA, Lu(III)-DOTA or In(III)-DOTA compounds [23], which is in contrast to what has been observed for the agonists [17,24]. Interestingly, however, substitution of DOTA by the NODAGA chelator in the antagonists was able to increase massively the binding affinity of the Ga(III)-NODAGA analogs, compared with the Ga(III)-DOTA analogs. From the preclinical studies the sstr2-antagonist JR11 has been selected for clinical development. In the literature the DOTA-conjugate (DOTA-JR11) is also referred as OPS201 and the NODAGA-conjugate (NODAGA-JR11) is also referred as OPS202.

Five in vivo studies in different tumor models comparing head-to-head sstr2-antagonists with agonists have been performed. In four out of five it was shown that the antagonists bind to sstr2-expressing tumors in vivo better than the agonists with comparable or even higher affinity [14,23,25,26,27,28]. Only one out of these studies did not confirm the higher uptake of the antagonist [19]. Among these in vivo preclinical studies the comparison of the antagonist DOTA-JR11 with the state-of-the-art agonist DOTA-TATE is worth mentioning: (a) ^68^Ga-DOTA-JR11 having a dramatically lower affinity for the sstr2 (~150-fold) showed a 1.3-fold higher tumor uptake, while ^68^Ga-NODAGA-JR11 with a 6-fold lower affinity showed an up to 1.7-fold higher tumor uptake [23]; (b) treatment using ^177^Lu-DOTA-JR11 compared with ^177^Lu-DOTA-TATE resulted in longer tumor growth delay time and longer median survival for mice treated with the antagonist [25]; (c) ^177^Lu-DOTA-JR11 had a higher tumor residence time, compared with ^177^Lu-DOTA-TATE, with an impressive effect of its specific activity on the in vivo uptake and dosimetry that might further improve the safety window of PRRT with antagonists [26,27,28].

### 2.2. In Vitro Human Data

The first in vitro studies in human tumors confirmed the in vitro and in vivo data discussed above. In about fifty different human sstr2-positive successive tumor sections ^177^Lu-DOTA-BASS had higher binding capacity than ^177^Lu-DOTA-TATE, with mean ratios of antagonist-to-agonist ranged from 4.2 up to 12.3, as shown using in vitro receptor autoradiography [29]. More importantly, the sstr2-antagonist JR11 labeled with ^125^I was compared with ^125^I-TOC (both having similar affinity) in vitro, in a wide range of non-neuroendocrine tumors, such as cancers from prostate, breast, colon, kidney, thyroid, and lymphoid tissues [30]. It was shown that ^125^I-JR11 had a much higher binding capacity in renal cell cancers, breast cancer, medullary thyroid cancers and non-Hodgkin lymphomas than ^125^I-TOC, reaching levels that may be detected more easily in vivo than with current agonists. This makes radiolabeled sstr2-antagonists particularly attractive for targeting these tumors. 

### 2.3. Clinical Achievements

**Figure 2:**
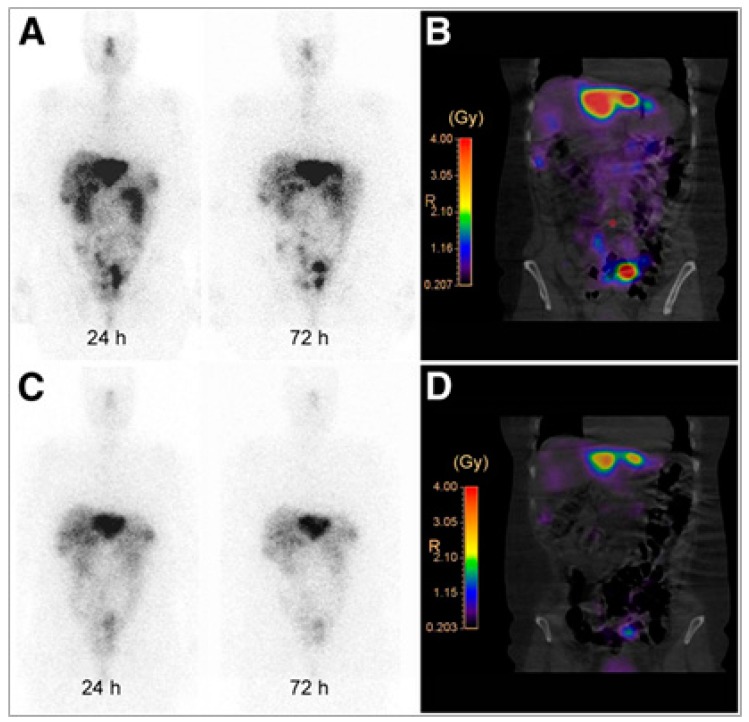
Comparison ^177^Lu-DOTA-JR11 and ^177^Lu-DOTA-TATE in a female patient with neuroendocrine tumor of the ileum. The figure presents planar scans and isodose curves after administration of 850 MBq of ^177^Lu-DOTA-JR11 (**A**, **B**) and 990 MBq of ^177^Lu-DOTA-TATE (**C**, **D**). Planar scans (**A**, **C**) show results 24 and 72 h after injection of ^177^Lu-DOTA-JR11 and ^177^Lu-DOTA-TATE. This research was originally published in *JNM*. Reproduced from [32]. © By the Society of Nuclear Medicine and Molecular Imaging, Inc.

The first clinical evidence that radiolabeled sstr2 antagonists can be used for in vivo targeting of sstr2 was provided from a pilot imaging study comparing^111^In-DOTA-BASS with Octreoscan^TM^ in five patients with metastatic thyroid carcinoma or neuroendocrine tumors [31]. ^1^^11^In-DOTA-BASS imaging resulted in a higher tumor detection rate due to its favorable biodistribution profile, summarized in up to 4.1 times higher tumor uptake and lower uptake in organs such as liver and spleen 4 h after administration, when compared with Octreoscan^TM^. Soon after, the same group provided the first clinical evidence that not only imaging but also treatment of NENs is feasible with radiolabeled sstr2-antagonists, in particular ^177^Lu-DOTA-JR11 [32]. In this pilot study ^177^Lu-DOTA-JR11 was compared with the agonist ^177^Lu-DOTA-TATE (Figure 2) in the same four patients, and showed a favorable pharmacokinetic and increased tumor dose due to a longer intra-tumoral residence time and a higher tumor uptake. Regarding dosimetry, the effective dose of ^177^Lu-DOTA-JR11 was slightly higher when compared with ^177^Lu-DOTA-TATE (0.20±0.0075 vs 0.15±0.0046 mSv/MBq), and also the kidneys and red marrow doses were higher for the antagonist. However, tumor-to-kidney and tumor-to-bone marrow dose ratios, the two most critical ratios for PRRT, were up to 6.2 and 7.2 times higher for the antagonist.

Recently, a phase I/II study comparing two microdoses (15 and 50 µg) of ^68^Ga-NODAGA-JR11 (^68^Ga-OPS202) with ^68^Ga-DOTA-TOC PET/CT, in the same patient, was conducted at the University Hospital of Basel (Basel, Switzerland; ClinicalTrials.gov NCT02162446, sponsored by Octreopharm/Ipsen). The study indicated increased image contrast for both doses of ^68^Ga-NODAGA-JR11, due to lower hepatic, intestinal and pancreatic uptake. This, in turn, resulted in a higher sensitivity and diagnostic accuracy (overall and especially for detecting liver metastases) than ^68^Ga-DOTA-TOC PET/CT for staging well to moderately differentiated GEP-NET patients [26,33]. The dosimetry of ^68^Ga-NODAGA-JR11 has not yet been published, however the respective total effective dose is expected to be on the level of such sstr agonists as ^68^Ga-DOTA-TOC and ^68^Ga-DOTA-TATE (0.021±0.003 mSv/MBq [34]). The “theranostic pair” ^68^Ga-DOTA-JR11 and ^177^Lu-DOTA-JR11 is currently evaluated in NEN patients at Memorial Sloan Kettering Cancer Center (New York, NY, USA; ClinicalTrials.gov NCT02609737). Larger-scale multicenter clinical trials are in preparation for both, ^68^Ga-NODAGA-JR11 and ^177^Lu-DOTA-JR11.

## 3. Glucagon-Like Peptide-1 Receptor Targeting

GPL-1R is a seven-transmembrane topology receptor overexpressed in such pathologies as insulinomas, gastrinomas, and phaeochromocytomas [35,36,37,38]. It is also expressed physiologically in the endocrine pancreas, intestine, lung, kidney, breast and brain [36]. Insulinoma is the most common form of pancreatic NENs of beta-cell origin and the most common cause of hyperinsulinemic hypoglycemia [39,40,41]. Most insulinomas are benign and single, and less than 10% are malignant and multiple [38]. The sstr density is low in benign insulinomas even though they are neuroendocrine tumors, whereas GLP-1R is expressed with high incidence and density [36,37,42,43,44]. Malignant insulinomas express sstr in density adequate to the imaging and GLP-1R to much lesser or non-existent extent [45]. However, both SPECT and PET clinical studies demonstrated imaging of malignant insulinoma using exendin-4 analogs [46,47,48]. Moreover, negative scan using imaging agents comprising GLP-1 analogs may potentially indicate malignancy. Thus it is recommended to performed combination of GLP-1R and sstr2 imaging for the diagnosis of insulinomas. The first line treatment for benign insulinomas is surgical excision however the small size makes their precise localization difficult [38,49]. Given the high in vivo sensitivity, resolution and sub nanomolar agent concentration, PET and SPECT are valuable techniques for the imaging of small lesions. A number of imaging agents for SPECT [50,51,52,53,54,55,56,57,58] and PET [46,57,58,59,60,61,62,63,64,65,66,67,68,69,70,71,72,73,74,75,76,77] have been developed to target GLP-1R.

### 3.1. Clinical Achievements

A number of clinical research studies has been performed since the first publication on 2 patients with insulinoma using [Lys^40^(Ahx-DTPA-^111^In)NH_2_]-exendin-4 for the imaging of GLP-1R [53] and at present several clinical research studies and multicenter clinical trials using various GLP analogs are ongoing in Europe [45,46,53,54,55,64,65,78]. Exendin-4 analogs that are relatively stable agonists of GLP-1R labeled with γ emitting radionuclides such as ^111^In and ^99m^Tc demonstrated high sensitivity in GLP-1R imaging and insulinoma detection with SPECT [54,55,78]. PET technique offers further advantages of higher sensitivity and spatial resolution as well as accurate quantification. These advantages are crucial especially considering the small size of insulinomas. Such β^+^ emitting radionuclides as ^18^F, ^64^Cu, ^68^Ga and ^89^Zr have been used presenting both advantages and drawbacks of their physical and chemical characteristics.

Exendin-4 ligand modified with either DTPA or DOTA via aminohexanoic acid (Ahx) spacer at a lysine amino acid residue and labeled with ^111^In, resulting in [Lys^40^-(Ahx-DTPA-^111^In)NH_2_]-exendin-4 and [Lys^40^-(Ahx-DOTA-^111^In)NH_2_]-exendin-4 demonstrated prominent detection of insulinomas that could not be unambiguously localized by conventional methods [53,55]. The examination enabled the surgeon to localize and resect the tumor in both patients [53]. Benign insulinomas were also detected in all six examined patients enrolled in [55] whereas conventional methods were conclusive only in four cases. Moreover, the long physical half-life of ^111^In allowed subsequent resection of the tumor mass by radioguided surgery using γ-probe intraoperatively [55]. The overexpression of GLP-1R in the resected lesion tissue was confirmed by in vitro autoradiography. The potential of [Lys^40^-(Ahx-DTPA-^111^In)NH_2_]-exendin-4 SPECT/CT for the improved patient management was investigated in a prospective study with eleven patients affected by malignant insulinoma [45]. The patients were also examined with ^68^Ga-DOTA-TATE PET/CT for the detection of sstr and the authors concluded that in contrast to benign insulinomas, malignant insulinomas often lack GLP-1R but express sstr2 more often. A subsequent larger study with thirty patients demonstrated that [Lys^40^-(Ahx-DTPA-^111^In)NH_2_]-exendin-4 SPECT/CT was more sensitive diagnostic technique than CT/MRI in detection of insulinomas and it changed therapeutic management of patients affected by endogenous hyperinsulinaemic hypoglycemia [78]. These successful studies also pointed out the limitation of the low spatial resolution of ^111^In/SPECT and interference of the high kidney uptake with detection of lesions in the pancreas. The adequate localization required a second SPECT examination 3–7 days later after the sufficient clearance of the kidneys from the radioactivity.

The lower γ-energy and shorter half-life of ^99m^Tc, as compared to ^111^In, could improve the quality of images and reduce radiation burden to the patient and medical staff. The ready availability of ^99m^Tc from a generator system provides another crucial advantage. The respective agent, [Lys^40^(Ahx-HYNIC-^99m^Tc/EDDA)NH_2_]-exendin-4, was used in a study of eleven patients with negative results on conventional diagnostic imaging methods [54]. The sensitivity and specificity of [Lys^40^(Ahx-HYNIC-^99m^Tc/EDDA)NH_2_]-exendin-4 SPECT/CT were assessed to be 100% in patients with benign insulinoma. In one patient out of two with malignant insulinoma the lesion was found in the region of local recurrence. In the subsequent study [48] forty patients with hypoglycemia were examined with [Lys^40^(Ahx-HYNIC-^99m^Tc/EDDA)NH_2_]-exendin-4 SPECT/CT and positive results were observed in twenty eight patients. The high kidney uptake presented similar complications as in the case of ^111^In-labeled analogs and the optimal imaging time in terms of pancreatic lesion localization was determined as 5-6 h post injection. [Lys^40^(Ahx-HYNIC-^99m^Tc/EDDA)NH_2_]-exendin-4 SPECT/CT was also successfully used for the diagnostic detection of medullary thyroid cancer [79].

**Figure 3:**
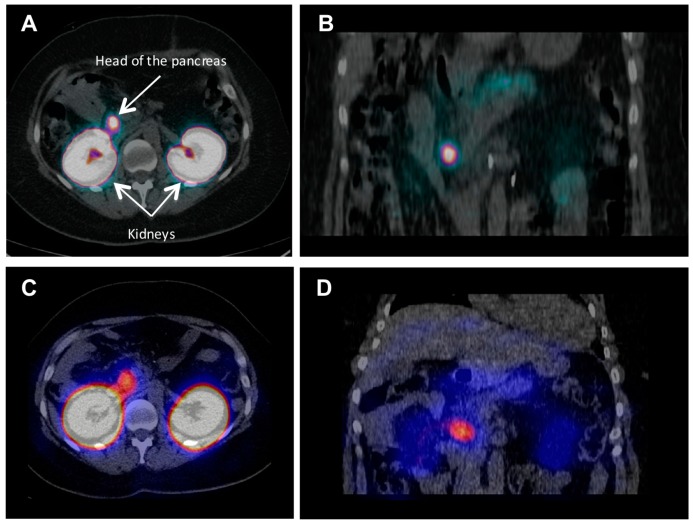
(**A**) Transaxial and (**B**) Coronal PET/CT images obtained 2.5 h after injection of 80 MBq [Nle^14^,Lys^4^^0^(Ahx-DOTA-^68^Ga)NH_2_]-exendin-4; (**C**) Transaxial and (**D**) Coronal SPECT/CT images of the same patient obtained 72 h after injection of 92 MBq [Nle^14^,Lys^4^^0^(Ahx-DOTA-^111^In)NH_2_]-exendin-4. Focal uptake is seen in the head of the pancreas. Courtesy of Dr. Kwadwo Antwi, University Hospital of Basel, Switzerland.

As already mentioned, PET technique offers advantages over SPECT in terms of higher spatial resolution and sensitivity, accurate quantification of the tracer uptake and consequently target concentration as well as possibility for dynamic scanning and subsequent kinetic modeling and uptake mechanism investigation. In particular, ^68^Ga is a very attractive radionuclide in terms of its ready availability from a simple generator system, straightforward labeling chemistry, and favorable decay characteristics [80]. A case examination of a patient with severe hypoglycemia was conducted using [^68^Ga]-DO3A-VS-Cys^40^-exendin-4 PET/CT [46]. Multiple small liver metastases and para aortal lymph node lesions were clearly visualized, while computed tomography, ultrasound, [^18^F]FDG/PET-CT or [^11^C]HTP/PET-CT, and Octreoscan^TM^ could not provide conclusive results. A clinical study, wherein five patients with endogenous hyperinsulemic hypoglycemia were enrolled, compared [Nle^14^,Lys^4^^0^(Ahx-DOTA-^111^In)NH_2_]-exendin-4 and [Nle^14^,Lys^4^^0^(Ahx-DOTA-^68^Ga)NH_2_]-exendin-4 in terms of detection rate, resolution, and background uptake [65]. [Nle^14^,Lys^4^^0^(Ahx-DOTA-^68^Ga)NH_2_]-exendin-4 PET/CT correctly identified the insulinoma in four of four patients, whereas [Nle^14^,Lys^4^^0^(Ahx-DOTA-^111^In)NH_2_]-exendin-4 SPECT/CT correctly identified insulinomas in two of four patients. [Nle^14^,Lys^4^^0^(Ahx-DOTA-^68^Ga)NH_2_]-exendin-4 was shown to be sensitive in localizing hidden benign insulinomas, and was found superior in terms of shorter examination time, higher tumor-to-background ratio, higher spatial resolution, lower radiation dose, and accurate quantification. Representative comparative examination images of this on-going phase II/III clinical trial (ClinicalTrials.gov NCT02127541) are shown in Figure 3 clearly demonstrating superiority of the ^68^Ga-labeled counterpart. The detection of occult insulinoma was enabled by ^68^Ga-NOTA-MAL-Cys^4^^0^-exendin-4 PET/CT and subsequent surgical removal of the pancreas tail insulinoma resulted in recovery from hypoglycaemia [64]. The imaging was performed 2 h post injection in order to decrease the kidney uptake and allow visualization of the pancreas tail. In the subsequent prospective study the authors explored the potential of ^68^Ga-NOTA-exendin-4 PET/CT for the detection of insulinomas in a larger patient cohort and found sensitivity in the localization of the lesions of 97.7% which was considerably higher than that of CT (74.4%), MRI (56.0%), EUS (84.0%), and ^99m^Tc-HYNIC-TOC (19.5%) [47]. The lesions as small as less than 1.0 cm were detected by ^68^Ga-NOTA-MAL-Cys^40^-exendin-4 PET/CT in eleven patients. The high kidney uptake was high interfering with the detection of pancreas tail lesions, however additional examination 2–3 h post injection resulted in unambiguous delineation. Noteworthy, the only patient diagnosed with malignant insulinoma showed high uptake in both ^68^Ga-NOTA-MAL-Cys^40^-exendin-4 PET/CT and ^99m^Tc-HYNIC-TOC.

To our best knowledge the published patient studies did not present any cases of adverse effect such as tachycardia even though pigs developed severe tachycardia upon administration of [^68^Ga]-DO3A-VS-Cys^40^-exendin-4 [81,82]. In one patient only transient palpitation at the time of injection that lasted a few seconds has been reported [47].

### 3.2. Dosimetry and Feasibility of Radiotheranostics

The assessment of potential radiotoxicity to the essential radiosensitive organs, such as bone marrow, organs with physiological uptake of the radiopharmaceutical and healthy tissue surrounding lesions as well as excretory organs is of outmost importance for both diagnostic and radiotherapeutic agents. Moreover, pre-therapeutic dosimetry might allow for the dose planning of safe and effective internal radiotherapy wherein under treatment as well as nephrotoxicity can be avoided. One of the main shortcomings of the clinically used agonist exendin analogs is high kidney uptake resulting in high radiation dose and compromising lesion detection in the adjacent head and tail of the pancreas [46,55,64]. 

Lower radiation burden from ^68^Ga-labeled analogs as compared to ^64^Cu [68] and ^111^In [58] labeled counterparts was demonstrated pre-clinically. The dosimetry of [^68^Ga]-DO3A-VS-Cys^40^-exendin-4 was investigated in various species demonstrating high kidney uptake in rats, pigs, non-human primates and a human with respective high absorbed doses [46,67,81,83]. Still, it would allow for 2–6 PET examinations in a human per year that might be sufficient for treatment response monitoring. The dosimetric calculations were also conducted by extrapolation of [^177^Lu]-DO3A-VS-Cys^40^-exendin-4 organ distribution performed in rats [84]. Preliminary estimation of tumor absorbed dose, based on a case examination of a patient affected by insulinomas using [^68^Ga]-DO3A-VS-Cys^40^-exendin-4 [46], demonstrated necessity for kidney protection and tumor accumulation enhancement in order to enable the use of [^177^Lu]-DO3A-VS-Cys^40^-exendin-4 for internal radiotherapy.

The increase in the tumor uptake and administered peptide dose could potentially be achieved by the introduction of antagonist analogs. Exendin(9-39)-amide isolated from *Helodermasuspectum* venom have been studied preclinically [56,85,86], however the number of binding sites was not higher for the antagonist ^125^I-BH-exendin(9-39), as compared to the agonist [87], and the tumor uptake decreased by 50% within 4 h [85]. Another antagonist analog, [Lys^40^(DTPA-^111^In)]-exendin(9-39), was compared to the agonist agents [Lys^40^(DTPA-^111^In)]-exendin-3 and [Lys^40^(DTPA-^111^In)]-exendin-4 [56]. All three agents exhibited similar IC_50_ values in cell culture, however antagonist [Lys^40^(DTPA-^111^In)]-exendin(9-39) demonstrated low specific uptake with fast washout in vivo in mouse xenografts.

Preclinical studies have been conducted to investigate the kidney uptake mechanism [50] and to reduce the uptake using poly-glutamic acid and gelofusine [58,88]. The kidney uptake decrease could also be achieved using Ex(9-39)NH_2_ antagonist analogs labeled with non-residualizing ^125^I moiety [52,85,89] and ^18^F [63,71]. Given the low kidney uptake the pair of positron emitting ^124^I and radiotherapeutic ^131^I could work in the context of radiotheranostics [6]. However, the radioactivity in the mouse xenograft when using ^125^I-BH-Ex(9-39)NH_2_ was reduced by 50% after 4 h and by 98% after 24 h [85]. Thus, a dedicated study that would demonstrate the therapeutic effect despite fast radioactivity clearance from lesions must be conducted in order to prove the therapeutic potential of ^131^I-BH-Ex(9-39)NH_2_. 

The radiotherapeutic effect of [Lys^40^(Ahx-DTPA-^111^In)NH_2_]-exendin-4 was studied in a transgenic mouse model of human insulinoma demonstrating reduction of the tumor volume by up to 94% [90]. However, the high kidney uptake is the major hinder for the radiotheranostic application of ^111^In.

## 4. Cholecystokinin 2/Gastrin Receptor Targeting

The CCK2 receptor is (over)expressed in several tumor types, including NENs, such as medullary thyroid cancer (MTC) and gastro-entero-pancreatic tumors, but also small cell lung cancer (SCLC), astrocytomas, stromal ovarian cancer, breast, endometrial and ovarian adenocarcinomas [91,92]. MTC is a neuroendocrine neoplasia of the parafollicular or C cells of the thyroid. The management of MTC mainly relies on surgical resection; however, recurrent disease develops in approximately 50% of patients with MTC [93]. Long-term responses by radiotherapy or systemic therapy are uncommon [94]. Different anatomic and molecular imaging methods (e.g., PET with ^18^F-DOPA or ^18^F-FDG, SPECT with ^99m^Tc(V)-DMSA, ^99m^Tc-MIBI or ^123^I-MIBG and PET or SPECT with radiolabeled sst analogs) have been used to localize recurrence and metastases in patients with MTC [95,96]. Despite these numerous diagnostic tools, there are still many patients with negative result on imaging scans and elevated post-operative calcitonin and short calcitonin doubling-time, often indicating unresectable distant metastases, including lung and liver [97].

MTC expresses two types of GPCR, the CCK2R and the sstr, as revealed by autoradiographic studies, followed by clinical investigations [22,23,24,25]. The expression of sstr in MTC is not as high as CCK2R (40% vs 90%) [22]. Moreover, the grade of differentiation of the MTC tumor cells is inversely related to the sstr expression. CCK2R scintigraphy showed to have an added value and improved tumor detection, in comparison to sstr scintigraphy [98,99,100].

### 4.1. Clinical Achievements

There are several reports on small scale clinical pilot-studies utilizing radiolabeled sulfated (s)CCK8 and non-sulfated (ns)CCK8 or minigastrin (MG) analogs for CCK2R targeting. In one of the first studies in MTC patients the nsCCK8 analogs DTPA/DOTA-D-Asp-Tyr-Nle-Gly-Trp-Nle-Asp-Phe-NH_2_ (Table 2), labeled with ^111^In, were used [101,102]. The tumor uptake and the tumor-to-background ratio were low, while intensive CCK2R-specific stomach uptake was found. The next generation analogs were based on MG (Table 2) labeled with ^131^I or ^111^In via DTPA [103,104]. Visualization of pathologically expressing CCK2R-positive lesions was achieved using ^111^In-DTPA-MG. The physiological uptake of ^111^In-DTPA-MG was restricted to the stomach (the organ with the physiologically highest expression of CCK2R) and the kidneys as the excretory organ. Imaging with ^111^In-DTPA-MG could favor the detection of liver metastases due to the considerably lower liver uptake, as observed with ^111^In-DTPA-OC [105]. The peptide sequence of ^111^In-DTPA-MG was later on modified to ^111^In-DTPA-[D-Glu^1^]-MG (MG0, Table 2) in order to improve the in vivo enzymatic stability of the radiotracer and consequently its clinical performance [106]. ^111^In-DTPA-MG0 scintigraphy was able to visualize all lesions, known from other imaging modalities, in a study involving seventy five patients with metastatic MTC [107], while in patients with occult disease the patient-based sensitivity was 91%.

The reduced liver and spleen uptake of the MG analogs, together with their improved affinity for the CCK2R, as compared to sCCK8 derivatives, favored their use as CCK2R-targeting probes [107]. Froeberg et al. reported the side-by-side comparison of the CCK8 analog ^111^In-DOTA-CCK8 (Table 2), the minigastrin analog ^99m^Tc-demogastrin 2 (Table 2) and the truncated MG analog ^111^In-DOTA-MG11 (Table 2) [110]. This comparison indicated that the full length minigastrin analog (^99m^Tc-demogastrin 2) appears to be the most promising diagnostic tool in patients with evidence of recurrence of metastases of MTC. In this small group of patients the CCK8 analog and the truncated minigastrin analog ^111^In-DOTA-MG11 performed worse, most probably due to their low enzymatic stability in vivo [110,111]. Behr and Behe were the first to use a therapeutic tracer targeting CCK2R, namely ^90^Y-DTPA-MG0 in patients with advanced metastatic MTC [107]. Eight patients were injected with potentially therapeutic doses of ^90^Y-DTPA-MG0 at 4–6-weekly intervals in an escalation study. Higher therapeutic efficacy seemed to be associated with higher dose levels. Unfortunately, the first experience with PRRT resulted in rather severe nephrotoxicity [107]. Therefore, a number of pre-clinical studies focused on reducing the kidney uptake of this class of radiopharmaceuticals [88,110,111,113,117,118,123]. High kidney retention of the MG analogs was related to the N-terminal ionic glutamic acid residues and could be substantially reduced by co-injection of poly-glutamic acids [123] or the gelatin-based plasma expander gelofusine [88,117,118]. Using a chemical approach, by elimination of the charged glutamic acid residues, the group of Maecke demonstrated a positive correlation between the low kidney uptake and retention and the reduced number of negative charges [111]. The deletion of five glutamic acid residues in the sequence of MG0 (resulting in MG11, Table 2) led to a reduction of the accumulation in the kidneys by a factor of more than 25 and subsequently to a significant improvement of the tumor-to-kidney ratio [111]. Similar results were observed with the deletion of the penta-glutamate sequence from MG0 and insertion of histidine residues H2Met/H2Nle (Table 2) [113]. While kidney uptake was considerably decreased at the same time the tumor uptake and the enzymatic stability were compromised and the analogs were found not favorable for clinical use [110]. 

The problem of low in vivo stability was addressed by further chemical modifications employing different approaches, such as cyclisation (Cyclo-MG1, Table 2), polyvalency (MGD5, Table 2) or introduction of different non-ionic D-amino acid residues (PP-F10, Table 2) [114,115,116]. Unexpected were the findings of introducing ionic D-amino acid residues (PP-F11, PP11, CP04, MG48, Table 2) resulting to favorable pharmacokinetics, high metabolic stability and good tumor retention, despite the hypothesis that the charge of the peptide analog determines its retention in the kidneys. In a comprehensive comparative study, within the European COST action BM0607, among 12 tested radiolabeled analogs targeting CCK2R the analog PP-F11 (CP04) was described as one of the most promising MG analogs for clinical translation [118,120,121,122,124,125]. A case-report using ^68^Ga-labeled DOTA-PP-F11 (CP04) in a patient with CCK2R-expressing MTC demonstrated its potential [126].

Currently, there are two on-going clinical studies on MTC patients with unresectable disease. The first study is a multicenter trial being implemented within the framework of a multinational European cooperation project on personalized medicine (TRANSCAN call of the EU within ERANET, project GRAN-T-MTC), where ^111^In-PP-F11 (CP04) is evaluated in MTC patients. The aim of the study is to establish the safety of intravenous administration of a therapeutic amount of the PP-F11 (CP04) peptide and to assess the biodistribution and dosimetry of ^111^In-PP-F11 (CP04). Only if safety is ensured later on the translation towards radionuclide therapy using e.g., ^177^Lu will be applied [118,127]. The second clinical trial is a pilot and a phase I study using ^177^Lu-PP-F11N (ClinicalTrials.gov NCT02088645), aiming in the evaluation of the scintigraphic visualisation of metastases and determination of the maximum tolerated dose (MTD) and adverse effects of ^177^Lu-PP-F11N treatment. The analog PP-F11N (Table 2) is a derivative of PP-F11 with isosteric replacement of Met by non-oxidazing amino acid residue Nle, resulting in circumventing the problem of the oxidation of Met, while allowing similar properties to PP-F11 (CP04), as shown by preclinical data [119,127]. Currently, no results from any of the two clinical studies are published. 

### 4.2. Dosimetry and Feasibility of Radiotheranostics

Since the initial PRRT attempt with ^90^Y-DTPA-MG0 resulted in rather severe kidney toxicity careful dosimetric estimation and potential therapeutic application of PP-F11 (CP04) were assessed in pre-clinical settings [107,118,124]. The limiting organ proved to be the excretory organs—the kidneys, followed by CCK2R-positive stomach wall. The absorbed doses for the kidneys and the stomach wall were 56 and 15 mGy/MBq, respectively for ^111^In-PP-F11 (CP04) and 400 and 86 mGy/MBq, respectively for ^177^Lu-PP-F11 (CP04) [118]. Based on dosimetric calculations from animal data the effective dose of ^111^In-PP-F11 (CP04) predicted for human was below 10 mSv (9.9 mSv/220 MBq), while the absorbed dose for the kidneys was 27.5 mGy/220 MBq. These are much lower than the ones estimated for Octreoscan^TM^, where according to SPC (Summaries of Product Characteristics) are 26 mSv/220 MBq and 108.3 mGy/220MBq, respectively [118].

In an extensive study by Konijnenberg et al. the influence of the particle range (^9^^0^Y, ^177^Lu, ^213^Bi), the activity and the peptide amount of PP-F11 (CP04) were studied [124]. It was found that increasing the amounts of peptide causes a saturation effect on the receptor-mediated uptake in the tumor, while having hardly any influence on the renal uptake, calling for careful assessment of the optimal peptide mass in human studies. Use of high peptide mass in order to increase the amount of radioactivity administered could result in lower tumor uptake and at the same time higher absorbed dose to the kidneys [124]. There is one more reason for careful selection of the peptide mass for eventual PRRT. All described small-scale clinical studies reported on side effects upon injection of the radiotracers. A peptide amount of 5–10 µg mostly caused a short episode of pentagastrin stimulation test-like symptoms. Administration of the radiotracers was often followed by a clear increase of serum calcitonin levels and was sometimes associated with mild side effects: increase of heart rate, flushes, mild nausea, paraesthesia in the hands and some dizziness. All side effects were mild and disappeared spontaneously within 10 min after the administration [110,128]. In the case of PRRT higher peptide masses are foreseen in order to administered therapeutic amounts of radioactivity. Practically, achievable maximum specific activities for PP-F11 (CP04) labeled with ^9^^0^Y and ^177^Lu were 400 MBq/nmol and 120 MBq/nmol, respectively [124].

Fractionation of the therapeutic doses is suggested as it may combine, from one side the practically achievable specific activities of the radiopharmaceuticals and on the other side the maximal peptide mass to be administered without causing saturation of the CCK2R or side effects. Moreover, it has been showed that fractionation leads to higher cumulative absorbed doses in tumors and lower probability of renal toxicity in clinical application of PRRT [129].

## 5. Glucose-Dependent Insulinotropic Polypeptide ReceptorTargeting

The targeting of the GIP, also known as Gastric Inhibitory Polypeptide, can represent a valid alternative to the use of the most common targeting agents for NENs based on somatostatin and GLP-1 analogs. GIP is a 42-amino acid peptide, synthesized by enteroendocrine K cells and it stimulates insulin secretion from pancreatic-cells after ingestion of nutrients. GIP exerts its function through the GIPR that belongs to the secretin receptor family, a subgroup of the GPCR family and it is expressed in low density everywhere in the body [130]. Recent studies demonstrated the high incidence and receptor density of GIP-R in pathologic condition, in a broad spectrum of human gastrointestinal and bronchial NENs. Interestingly, high overexpression of GIP-R is found in most of the sstr-negative and GLP-1R-negative NENs [131]. This finding opens new possibility for the use of GIP-based radiotracers as alternative or complementary to the well-established somatostatin-based ones. Due to the increasing interest for potential clinical application, the development of radiolabeled GIP-based analogs is emerging. The truncated peptide GIP(1-30), that activate the receptor with equal potency as the native GIP(1-42) [132], was used as model peptide by Gourni et al [133] to develop a new class of GIP based radioligands that can be potentially used for the in vivo targeting of GIP-R-positive tumors. Among the several analogs developed, ^111^In-EG4 ([Nle^14^,Lys^3^^0^(Ahx-DOTA)]GIP(1-30)NH_2_) showed favorable in vitro properties with good affinity and high and specific internalization rate in transfected INR1G9-hGIPr cell line (pancreatic endocrine cell line INR1G9 transfected with the human GIP receptor). Good and specific tumor visualization in INR1G9-hGIPr xenografts with the ^68^Ga-EG4, along with biodistribution data, confirm the feasibility of this approach for imaging GIPR-positive tumors. Further radiolabeled GIP analogs are currently under preclinical development and evaluation. 

## 6. Summary

Radiotheranostics using somatostatin analogs has become reality in the management of patients affected by neuroendocrine neoplasms. The radiolabeled somatostatin analogs have been the prototypes of peptide-based radiopharmaceuticals and helped creating an entire new field in oncology: peptide-receptor imaging and therapy. This paved the way for the development of various peptide-based radiopharmaceuticals for diagnosis and radionuclide therapy of various NENs targeting other receptors, in particular GLP-1R, CCK2, and GIP-R.

Recent developments challenge the use of radiolabeled somatostatin receptor agonists, used for more than 20 years. Radiolabeled somatostatin receptor antagonists have shown to outperform agonists, in terms of tumor uptake and retention. It remains to be seen if this new class of radiopharmaceuticals will prove to be superior to the agonist analogs used in the clinic currently. In the field of benign insulinomas, a challenging disease due to the small size of the lesions, several clinical multicenter diagnostic studies are ongoing in Europe using exendin-4 analogs targeting GLP-1R. However, further improvement of the agents in terms of reduction of kidney uptake and potential side effects induced by agonists is desirable. The clinical impact of radiolabeled gastrin analogs in MTC patients still remains to be proven, with the currently ongoing clinical studies focusing on theranostics. Last, but not least, targeting GIP receptors offers an option for sstr-negative and GLP-1R-negative NENs that are not covered by the existing specific-targeting radiopharmaceuticals. This approach is at a very early stage.

## Figures and Tables

**Table 1:** Amino acid sequence of the somatostatin analogs discussed in the review.

Code	Chemical Structure
**Somatostatin receptor agonists**	
OC	d-Phe-cyclo(Cys-Phe-d-Trp-Lys-Thr-Cys)Thr(ol)
TOC	d-Phe-cyclo(Cys-Tyr-d-Trp-Lys-Thr-Cys)Thr(ol)
TATE	d-Phe-cyclo(Cys-Tyr-d-Trp-Lys-Thr-Cys)Thr
NOC	d-Phe-cyclo(Cys-1-Nal-d-Trp-Lys-Thr-Cys)Thr(ol)
**Somatostatin receptor antagonists**	
BASS	p-NO_2_-Phe-cyclo(d-Cys-Tyr-d-Trp-Lys-Thr-Cys)d-Tyr-NH_2_
LM3	p-Cl-Phe-cyclo(d-Cys-Tyr-d-Aph(Cbm)-Lys-Thr-Cys)d-Tyr-NH_2_
JR10	p-NO_2_-Phe-cyclo(d-Cys-Tyr-d-Aph(Cbm)-Lys-Thr-Cys)d-Tyr-NH_2_
JR11	p-Cl-Phe-cyclo(d-Cys-Aph(Hor)-d-Aph(Cbm)-Lys-Thr-Cys]-d-Tyr-NH_2_

1-Nal = 1-naphthyl-alanine; Aph(Hor) = 4-amino-l-hydroorotyl-phenylalanine; d-Aph(Cbm) = d-4-amino-carbamoyl-phenylalanine.

**Table 2:** Cholecystokinin 8 (CCK8) and minigastrin analogs and conjugates.

Code	Chemical Structure	Reference
***CCK8 analogs***		
CCK8	d-Asp-Tyr-Met-Gly-Trp-Met-Asp-Phe-NH_2_	
sCCK8	d-Asp-Tyr(OSO_3_H)-Met-Gly-Trp-Met-Asp-Phe-NH_2_	
CCK8(Nle)	d-Asp-Tyr-**Nle**-Gly-Trp-**Nle**-Asp-Phe-NH_2_	P: [108,109]; C: [101,102,110]
***Minigastrin analogs***		
MG	Leu^1^-Glu^2^-Glu^3^-Glu^4^-Glu^5^-Glu^6^-Ala^7^-Tyr^8^-Gly^9^-Trp^10^-Met^11^-Asp^12^-Phe^13^-NH_2_	P, C: [105]
MG0	**d-Glu^1^**-Glu^2^-Glu^3^-Glu^4^-Glu^5^-Glu^6^-Ala^7^-Tyr^8^-Gly^9^-Trp^1^^0^-Met^11^-Asp^12^-Phe^13^-NH_2_	P: [106]; C: [99,107]; CP
MG11	**d-Glu**-Ala-Tyr-Gly-Trp-Met-Asp-Phe-NH_2_	P: [109,111]; C: [98,110]; CP
Demogastrin 2 (N_4_-conjugate)	N_4_-**Gly-d-Glu**-(Glu)_5_-Ala-Tyr-Gly-Trp-Met-Asp-Phe-NH_2_	P: [112]; C: [103]
H2-Met, APH070	**His-His**-Glu-Ala-Tyr-Gly-Trp-Met-Asp-Phe-NH_2_	P: [113]; CP
Cyclo-MG1 (DOTA-conjugate)	DOTA-**DGlu**-(Ala-Tyr)-**d-Lys**-Trp-Met-Asp-Phe-NH_2_(cycloDGlu-DLys)	P: [114]; CP
MGD5 (divalent; DOTA-conjugate)	DOTA-**Gly-Ser-Cys**-(Glu-Ala-Tyr-Gly-Trp-**Nle**-Asp-Phe-NH_2_)_2_	P: [115]; CP
PP-F10 (DOTA-conjugate)	DOTA-(**d-Gln**)_6_-Ala-Tyr-Gly-Trp-Met-Asp-Phe-NH_2_	P, C: [116]; CP
PP-F11 (DOTA-conjugate)	DOTA-(**d-Glu**)_6_-Ala-Tyr-Gly-Trp-Met-Asp-Phe-NH_2_	P: [117,118]; CP
		C: *in clinical trial*
PP-F11-N (DOTA-conjugate)	DOTA-(**d-Glu**)_6_-Ala-Tyr-Gly-Trp-**Nle**-Asp-Phe-NH_2_	P: [119]
		C: *in clinical trial*

P—radiopeptide tested in preclinical studies; C—radiopeptide administered to humans (clinical study); CP—COST peptide, evaluated in comparative studies [120,121,122].

## Abbreviations

The following abbreviations are used in this manuscript:

5-HTP	5-Hydroxy-l-tryptophan
Ahx	Aminohexanoic acid
BFC	Bifunctionalchelator
BH	Bolton-Hunter
CCK2(R)	Cholecystokinin 2 (Receptor)
CB-TE2A	4,11-Bis(carboxymethyl)-1,4,8,11-tetraazabicyclo[6.6.2]hexadecane
CT	Computed tomography
DMSA	Dimercaptosuccinic acid
DOPA	l-Dihydroxyphenylalanine
DOTA	1,4,7,10-Tetraazacyclododecane-1,4,7,10-tetraacetic acid
DTPA	Diethylenetriaminepentaacetic acid
EDDA	Ethylenediamine-*N*,*N*′-diacetic acid
FDA	Food and Drug Administration
FDG	Fluorodeoxyglucose
GEP-NET	Gastroenderopancreatic neuroendocrine tumors
GIP(R)	Glucose-dependent insulinotropic polypeptide (Receptor)
GIST	Gastrointestinal stromal tumors
GLP-1(R)	Glucagon-like peptide-1 (Receptor)
GPCR	G protein-coupled receptors
HEK	Human embryonic kidney
HYNIC	Hydrazinonicotinamide
MIBG	Meta-iodobenzylguanidine
MIBI	Methoxyisobutylisonitrile
MRI	Magnetic resonance Imaging
MTC	Medullary thyroid cancer
NENs	Neuroendocrine neoplasms
MTD	Maximum tolerated dose
NODAGA	1,4,7-Triazacyclononane,1-glutaric acid-4,7-acetic acid
NOTA	1,4,7-Triazacyclononane-1,4,7-triacetic acid
NOTA-MAL	NOTA mono-*N*-ethylmaleimide
PET	Positron emission tomography
PRRT	Peptide Receptor Radionuclide Therapy
SPECT	Single-photon emission computed tomography
SST(R)	Somatostatin (Receptor)
VS	Vinylsulfonyl
